# Net primary productivity but not its remote‐sensing proxies predict mammal diversity in Andean‐Amazonian rainforests

**DOI:** 10.1002/ecy.70059

**Published:** 2025-03-10

**Authors:** Kim L. Holzmann, Pedro Alonso‐Alonso, Yenny Correa‐Carmona, Andrea Pinos, Felipe Yon, Alejandro Lopera, Gunnar Brehm, Alexander Keller, Ingolf Steffan‐Dewenter, Marcell K. Peters

**Affiliations:** ^1^ Department of Animal Ecology and Tropical Biology, Biocenter University of Würzburg Würzburg Germany; ^2^ Institut für Zoologie und Evolutionsbiologie mit Phyletischem Museum Friedrich‐Schiller University Jena Jena Germany; ^3^ Faculty of Biology, Cellular and Organismic Networks LMU Munich Planegg‐Martinsried Germany; ^4^ Departamento de Ciencias Biológicas y Fisiológicas, Facultad de Ciencias e Ingeniería Universidad Peruana Cayetano Heredia Lima Peru; ^5^ Instituto de Medicina Tropical Universidad Peruana Cayetano Heredia Lima Peru; ^6^ Andes Amazon Fund Washington DC USA

**Keywords:** Andes, biodiversity patterns, diversity gradients, elevational gradients, energy‐richness hypothesis, large mammals, more‐individuals hypothesis, NPP, Peru, tropical mountains

## Abstract

Tropical forests are disappearing, but we have a limited understanding of the factors driving species coexistence in mammal communities of old‐growth forest ecosystems. The total energy that is bound by plants is assumed to be a key factor determining mammalian species richness, but accurately measuring energy flows in complex ecosystems is difficult, and most studies therefore rely on remote‐sensing‐based surrogates of net primary productivity (NPP). We monitored mammal species richness across three seasons using camera traps on 26 study plots along a forested, elevational gradient from 245 to 3588 m above sea level in southeastern Peru for which a unique dataset on field‐measured NPP exists. Using linear‐regression models and path analysis, we disentangled the effects of climate and NPP on the diversity of mammals, testing the predictions of the more‐individuals hypothesis, stating that energy availability drives the number of individuals and, thus, the number of coexisting species. We compared detailed field measurements of NPP with remote‐sensing products (MODIS NPP and MODIS NDVI). Mammal species richness, abundance, and biomass decreased in a negative exponential pattern with elevation. Field‐measured data on NPP, which was largely driven by temperature, was a strong predictor of both abundance and species richness, while remotely sensed proxies for NPP failed to accurately predict mammal diversity. Our study underpins the importance of field‐based ecosystem data and emphasizes the role of high primary productivity for maintaining diverse mammal communities, which is a particularly pressing issue in light of recent anthropogenic impacts on the Amazonian forest system.

## INTRODUCTION

One of the major ecological tasks is the understanding of the distribution of biodiversity on Earth. Although there are various hypotheses emphasizing environmental factors and energy availability to explain the spatial variation in the distribution of species diversity (Brown, 2014; Currie et al., 2004; Peters et al., 2016, 2019; Sun et al., 2020), the underlying mechanisms remain poorly understood, and the impacts of various environmental factors are entangled. This restricts our current ability to make predictions on the impact of environmental change on biodiversity. Tropical elevational gradients offer a natural laboratory to investigate patterns and drivers of biodiversity on a relatively small scale (Silveira et al., 2019).

Based on previous studies, mammalian species richness seems to be controlled by the energy available for the community in a specific region (Currie et al., 2004; Gebert et al., 2019; Hawkins et al., 2003; Storch et al., 2018). The most direct mechanism behind this relationship is energy limiting the number of individuals and species' population sizes, which in turn limits the number of species that can coexist in an area—termed the “more‐individuals hypothesis” (MIH) (Srivastava & Lawton, 1998). Following a strict version of the MIH, available energy, the total number of individuals (termed abundance hereafter) and species richness are all positively correlated, while the relationship between species richness and abundance is stronger than the relationship between species richness and energy. However, a broader view of the MIH highlights the more complex species population dynamics, where abundance at one trophic level may increase energy availability for higher trophic levels (Storch et al., 2018). The major challenge of testing the MIH is to get an accurate measurement of energy and disentangle the network of interlinked climate, energy, and biodiversity variables (Šímová & Storch, 2017).

Energy can be measured as net primary productivity (NPP), which in turn can be parameterized in various ways but with strong divergences in estimates (Šímová & Storch, 2017). Ideally, NPP can be assessed directly in the field. However, measurements of NPP are very labor‐intensive, especially in complex vegetation such as tropical rainforests, which is why these data have scarcely been collected in tropical environments (Storch et al., 2018). In ecological studies, products of remote‐sensing technology are therefore often used to estimate NPP. However, remote‐sensing products of NPP are based on many assumptions, and results can be confounded by cloud cover. A common surrogate for NPP is the Normalized Difference Vegetation Index (NDVI), which strongly correlates with photosynthesis and biomass (Gould, 2000). In a further development, remotely sensed NPP values were made available, based on a more complex model including climate, spectral, and land use parameters (Running, 2002). Their suitability to predict diversity has scarcely been compared with true, field‐measured NPP, especially in complex vegetation such as very dense, multilayered rain‐ and cloud forests (but see McCain et al., 2018).

Available energy within an ecosystem is mediated by climatic factors, mainly temperature, which is another key predictor determining the distribution of biodiversity along environmental gradients (Rahbek et al., 2019). Temperature can act indirectly through its effect on NPP and energy availability for consumers (Classen et al., 2015; Peters et al., 2019), but it can also have a direct impact by fostering evolutionary rates (Allen et al., 2006; Brown, 2014), or by thermal limitations (García‐Robledo et al., 2016).

Mammal diversity along elevational gradients has been primarily studied for small mammals, that is, below 1.2 kg (Bogoni et al., 2016; Jiménez et al., 2010; Patterson et al., 1989, 1998; Pinho et al., 2017), while studies on larger mammals are scarce. In small mammals, most studies found a unimodal diversity distribution along elevational gradients (McCain, 2005). The same pattern was found in one study of large mammals along an elevational gradient at Mount Kilimanjaro (Gebert et al., 2019), but elevational diversity gradients of large mammals have rarely been researched in other biogeographic regions (but see Mena & Pacheco, 2022; Pinho et al., 2017).

We studied the patterns and predictors of large‐mammal diversity along a continuous elevation gradient in southern Peru with forest habitat relatively undisturbed by humans from the lowland Amazon basin (245 m above sea level [asl]) to the tree line in the Andes (ca. 3500 m asl). This region has been described as one of the most biologically diverse areas in the world (Patterson et al., 2006), and since 2003, it has been the long‐term study area of the “ABERG” Andes Biodiversity and Ecosystem Research Group (Fraser, 2023). ABERG conducts detailed field measurements of canopy litterfall, stem growth measurements, fine and coarse root production, and several other processes on one‐hectare plots to quantify the total NPP of tropical forest ecosystems in elevations from 120 to 3537 m asl (Malhi et al., 2017). This unique dataset allows testing the energetic limitations of mammalian species assemblages along a broad climate gradient with direct field measures of NPP in a relatively undisturbed tropical forest ecosystem.

We made the following hypotheses:

1. Mammal abundance, biomass, and the species richness of large mammals follow gradients of NPP, that is, decrease from low to high elevations.
2. The relationship between NPP and abundance is stronger than that between NPP and species richness.
3. Field‐measured NPP is a better predictor of mammalian diversity than remote‐sensing‐based estimates.

## MATERIALS AND METHODS

### Study area

**FIGURE 1:**
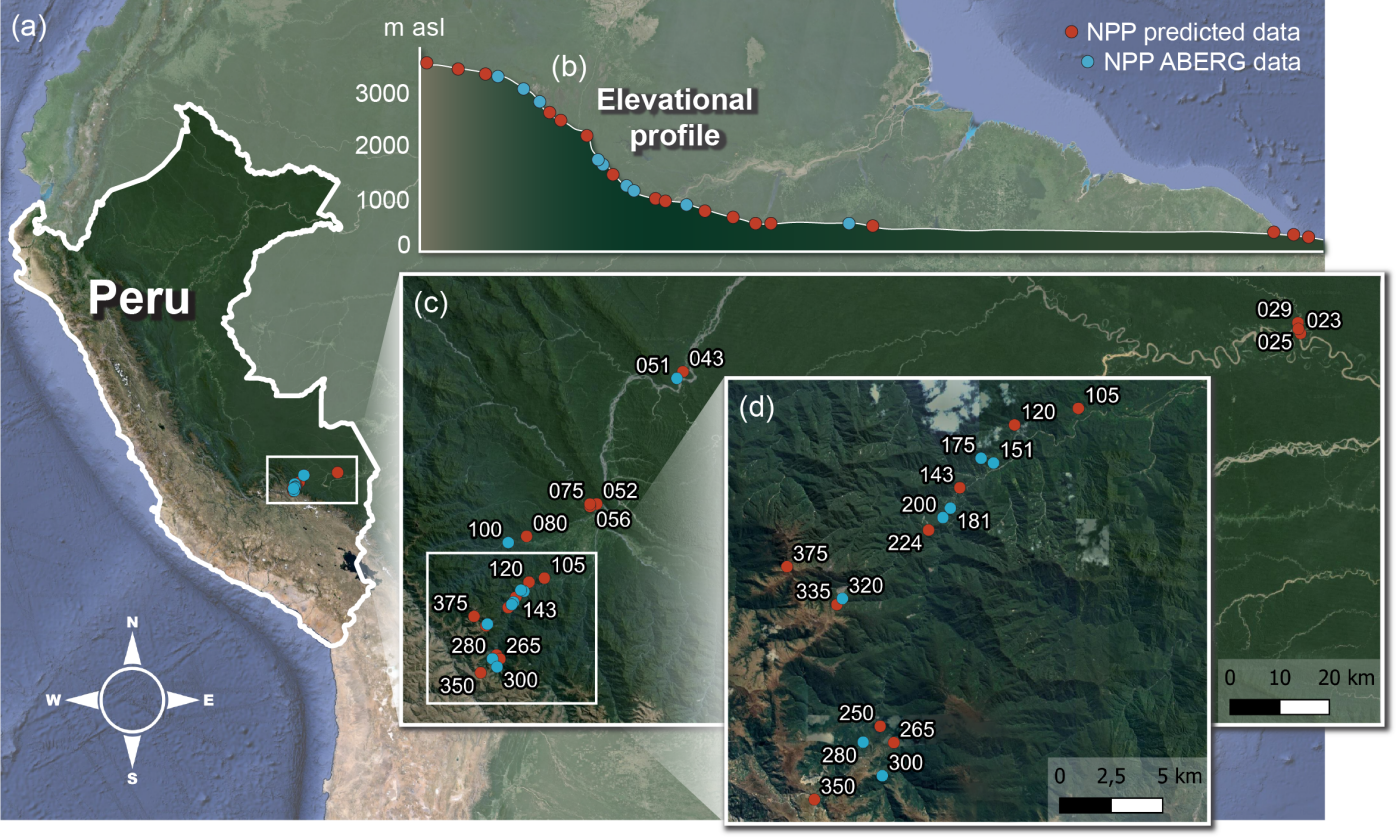
(a) Map of the study area in Peru and (b) the elevational profile of the studied gradient ranging from 245 to 3588 m above sea level, and (c) including 26 study plots in approximately 250‐m elevational intervals. (d) Zoomed‐in view of the steepest part of the gradient. Blue dots indicate positions of study plots for which long‐term net primary productivity (NPP) data was measured by the research group ABERG. Red dots indicate study plots for which NPP data were estimated from the long‐term field data.

This study was carried out along an elevational gradient in the Kosñipata valley in southeast Peru. The elevation gradient ranges from the Amazon basin at 245 m asl to the tree line at 3588 m asl (Figure 1). This gradient is continuously forested (wet rainforest, cloud forest) and habitat is mostly undisturbed by humans inside and in the buffer zone of the 18,812 km^2^ large Manu National Park.

The climate along the elevation gradient is characterized by three seasonal periods: a wet season from November through March, a dry season from May through July, and austral spring (steeper lapse rate, declining light, and vapor pressure deficit with altitude) in September and October (Rapp & Silman, 2012), with transitions in the months in between. Mean annual temperatures (MATs) ranged from 24.3°C in the lowlands to 6.7°C at 3600 m asl (tree line) and decreased linearly by approximately 4.4°C/1000 m elevation (2022–2023, method of temperature measurements below). Mean annual precipitation levels in the period 1998–2012 were high, with >1500 mm per year along the whole gradient, and a peak of ~5000 mm at around 1500 m asl (Malhi et al., 2017).

Research was conducted on 26 study plots, of which four were located inside Manu National Park. Study plots were roughly distributed in 250‐m elevation intervals along the Manu Road, the only access to the valley, and in the lowlands following the Madre de Dios River. Linear distances between plots were, on average, 52 km, with a mean distance of neighboring plots of 1519 m (minimum distance of a pair of plots: 408 m). The terrain in the study region is very complex, and many plots were additionally disconnected by steep valleys or rivers. Each study plot was investigated three times, once in each of the three seasonal periods (no surveys during the strongest rain season between January and March). Nine of the 26 study plots matched the research plots of the ABERG project (Andes Biodiversity and Ecosystem Research Group), providing long‐term field measurements of net primary productivity and other environmental data (see below).

### Climate data

Two types of temperature sensors were installed in all plots. One TMS‐4 soil and air temperature logger (TOMST s.r.o., Prague, Czech Republic) was positioned in each plot to record air temperature at 15 and 2 cm above the ground, as well as soil temperature and moisture at −6 cm depth (Wild et al., 2019). Measurements were recorded in 15‐min intervals, and sensors were collected after approximately 15 months (September 2022–December 2023) without any interference in between. For comparison, air temperature at 1.5 m height was additionally measured with iButton sensors (Analog Devices, Inc., Wilmington, USA) hanging from a horizontal branch in 240‐min intervals. The sensors were shielded with white plastic dishes (diameter ca. 18 cm) to protect them from rain and direct sunlight (Appelhans et al., 2016). Air temperatures at 1.5 m were highly predictive of the temperature at 15 cm height (*r* ^2^ = 0.999, *T*_air at 1.5 m_ = 0.26673 + 0.99845 × *T*_air at 15 cm_) and due to the denser sampling intervals, data from the TMS‐4 sensor were used for final statistical analyses. At one plot (ID “056”), the TMS logger could not be recovered due to fallen trees; therefore, the MAT from iButton data was used to predict *T*_air at 15 cm_ for this plot instead. MAT was calculated by averaging all temperature measurements across the sampling time. Mean annual precipitation (MAP) data for each plot was extracted from literature (Appendix S1; Huaraca Huasco et al., 2021).

### Measures of NPP

NDVI values were extracted for each plot using the AppEARS software (AppEEARS, 2024) and the C6 Moderate Resolution Imaging Spectroradiometer (MODIS) product MOD13Q1.061 (Didan, 2015) with 16‐day satellite data (available on 250‐, 500‐, and 1000‐m spatial resolution) over the whole study period. MODIS Terra was chosen, because of its morning orbit with more cloud‐free observations in the tropics in comparison to afternoon Aqua data (Lyapustin et al., 2014). To further avoid remaining cloud contamination, only pixels with high quality were kept, applying the pixel reliability scores (0 = “good data, use with confidence”). In final models, only the 250‐m radius was used, as it resulted in models with the highest level of explained variation and was more comparable to other studies (Classen et al., 2015; Mena & Pacheco, 2022). Mean values of NDVI (hereafter called MODIS NDVI) were calculated for the duration of the study year.

NPP was additionally extracted from satellite data using the MODIS product MOD17A3HGF.061 on a 500‐m spatial scale for the study year (in kilograms of C per square meter per year). MODIS NPP values were converted into megagrams of C per hectare per year. These estimates of NPP (hereafter called MODIS NPP) are based on a complex algorithm considering absorbed solar energy and its connection to remote‐sensing‐derived spectral indices of vegetation and calculated with the gross primary productivity and the maintenance respiration. For more information, see Running and Zhao (2015).

Field measures of NPP (in megagrams of C per hectare per year) done by ABERG were extracted from literature data (Malhi et al., 2017). ABERG measured NPP and allocations of NPP to different parts of plants (NPP allocated to canopy, leaves, herbivory, aboveground coarse wood productivity “ACW,” branch turnover, coarse root and fine root productivity) for a total of 14 study plots in the same study region. Nine of the 14 plots matched our study plots, and the remaining five plots were located in the same study area. Measurements followed the field protocol of the Global Ecosystems Monitoring network (Marthews et al., 2014). This protocol involves measuring canopy litterfall and estimating leaf loss to herbivory, scanning aboveground woody productivity of trees (>10 cm dbh), checking for freshly fallen tree branches, and ingrowth cores for root productivity (see details in Malhi et al., 2017). Using the NPP values from the ABERG project (Malhi et al., 2017), a predictive generalized additive model (gam) with elevation as a predictor variable was used to estimate NPP values for all plots without field‐measured NPP data (deviance explained = 72.3%, corrected Akaike information criterion [AIC_c_] = 68.68). As the trends in NPP are not driven by elevation per se but by elevational changes in temperature and precipitation, we additionally quantified the relationship between NPP (response variable), MAT, and MAP (predictor variables) in another gam, where MAT (effective df = 2.31, residual df = 21.69, *F* = 14.149, *p* < 0.001) was a significant predictor of NPP, but not MAP (effective df = 1.00, residual df = 21.69, *F* = 3.572, *p* = 0.08; Deviance explained = 80.5%, AIC_c_ = 73.85; Appendix S1: Figure S1). All statistical analyses were conducted with the data, including predicted NPP values (*n* = 26) and a data subset in which only data from study plots with field‐measured NPP data remained (*n* = 9).

### Mammal monitoring

Mammal data were collected by camera trap monitoring within each of the three 4‐month‐long field seasons: September to December 2022, April to August 2023, and September to December 2023. In each of the 26 plots, four camera traps (Bushnell Trophy Cam HD Essential) were attached to a tree at ~1 m height within a 20 m distance, if available near trails. Leaves and branches directly in front of the camera were removed. In each field season, cameras were operated for seven days, summing to 84 trap‐days per plot and 2184 trap‐days in total (i.e., 52,416 camera trap hours in total; 4 cameras × 7 days × 24 h × 3 field seasons). Activation worked with a motion sensor set to normal sensitivity level. At night, cameras operated with infrared light. They were programmed to record 10‐s videos with an inactive period of one minute. For analyses of large‐mammal relative abundance, we followed the hourly event count (Gebert et al., 2020; Hegerl et al., 2017), that is, two videos of the same mammal species were considered independent individuals if they were recorded with a time difference of at least one hour. Abundance, as measured in this study, should be interpreted with care, as higher abundances could be both due to more individuals living in the area or a higher activity of individuals in the area. All species were identified using the field guide from Emmons and Feer (1990) and taxonomy was updated according to the ASM database (ASM Mammal Diversity Database). Species were classified according to their body size as “small” with ≤1.2 kg, “medium” with >1.2 and ≤10 kg, and “large” mammals with >10 kg (Bogoni et al., 2016). Red list status was assessed (www.iucnredlist.org). Small rodents were identified on the morphospecies level, and the mass was based on the average of representative species of similar size (small rodents: average from *Mus musculus*, medium size rodents: average from *Rattus rattus*, mouse marsupials Didelphidae: average from all *Marmosa* sp. in database). In two recordings, a domestic dog was detected by the camera traps, and the average mass of a medium‐sized breed was assumed (20 kg). We included these two recordings in our analyses. Excluding the dog counts from the dataset did not lead to any changes in resulting patterns. All recorded species were grouped into the trophic guilds herbivores, omnivores, and carnivores (Emmons & Feer, 1990).

Abundance was estimated by summing the number of all individual records of each species (for each plot in each season; records of the four cameras within a single plot were pooled), and species richness by counting the number of recorded species. Assemblage biomass was calculated for each study plot by multiplying the average body mass retrieved from Wilman et al. (2014) of each species with its individual records and summing the species‐specific values across all species detected on a study plot. Both assemblage biomass and abundance were treated as comparative measures (in comparison to other study plots) rather than as a true measures of the total biomass in the study plot area.

### Statistical analysis

All statistical analyses were done in R 4.3.2 (R Core Team, 2023) and results were considered significant at α ≤ 0.05 (5%). For analyses of abundance, species richness, and biomass patterns along the gradient, generalized additive models (gam's; gaussian data) as implemented by the mgcv package were used to calculate elevational trends (Wood, 2011). A separate model was built for each field season (as well as for the cumulative dataset), for each trophic guild and for each size class. Residuals of the gam's were tested for spatial autocorrelation using Moran's *I* from DHARMa (Hartig, 2018), and no significant correlations were found (Appendix S2, Table S1).

To check sampling completeness per plot, the nonparametric Chao 1 was used to estimate asymptotic species richness (Colwell & Coddington, 1994) with the “estimate” function from the vegan package (Oksanen et al., 2022), and correlation to the observed species richness was checked with the Pearson correlation test. Using the observed elevational ranges (assuming a continuous distribution between minimum and maximum occurrence), the total number of species for each band of 250 m was calculated. Elevational trends were determined with a gam model as described above and compared to the pattern detected for the data based on 26 study plots.

Potential predictors for mammal abundance and species richness were assessed, first, with ordinary linear models and, second, by applying path analysis. The distribution of residual data was checked with quantile‐quantile plots and the Shapiro test for normality. Abundance and species richness data were log‐transformed to obtain a normal distribution of residuals. Correlations between all environmental variables were checked and visualized with “corrplot.” Next, field‐measured NPP, MODIS NDVI and MODIS NPP, as well as individual components of the field‐measured NPP (canopy, leaf, herbivory, ACW, branch turnover, coarse root, fine root) were tested individually as predictors of species richness and abundance in ordinary linear models. For multifactor models, two separate global models for abundance and species richness were created, each including MAT, MAP, and field‐measured NPP as explanatory variables. To compare species richness data to the estimated asymptotic number of species in the community, all analyses were additionally run with the Chao 1 estimated asymptotic species richness instead of the counted number of species (i.e., species richness). Multimodel inference was conducted by applying the “dredge” function from the MuMIn package, and the four models with the lowest AIC_c_ values were reported (Bartoń, 2023). The residuals of the best models were tested for spatial autocorrelation (Moran's *I*). Additionally, the best multifactor models and all NPP component analyses were also performed with a reduced dataset containing only the study plots on which NPP data was originally measured in the field by the ABERG project (excluding all plots for which NPP was predicted). Path analysis was applied to test the predictions of the more‐individual hypothesis, by using a standardized linear model (scale function) to check the relationships between NPP, abundance, and species richness. Additionally, Pearson correlation tests were used to make results comparable with literature data (Currie et al., 2004).

## RESULTS

**FIGURE 2:**
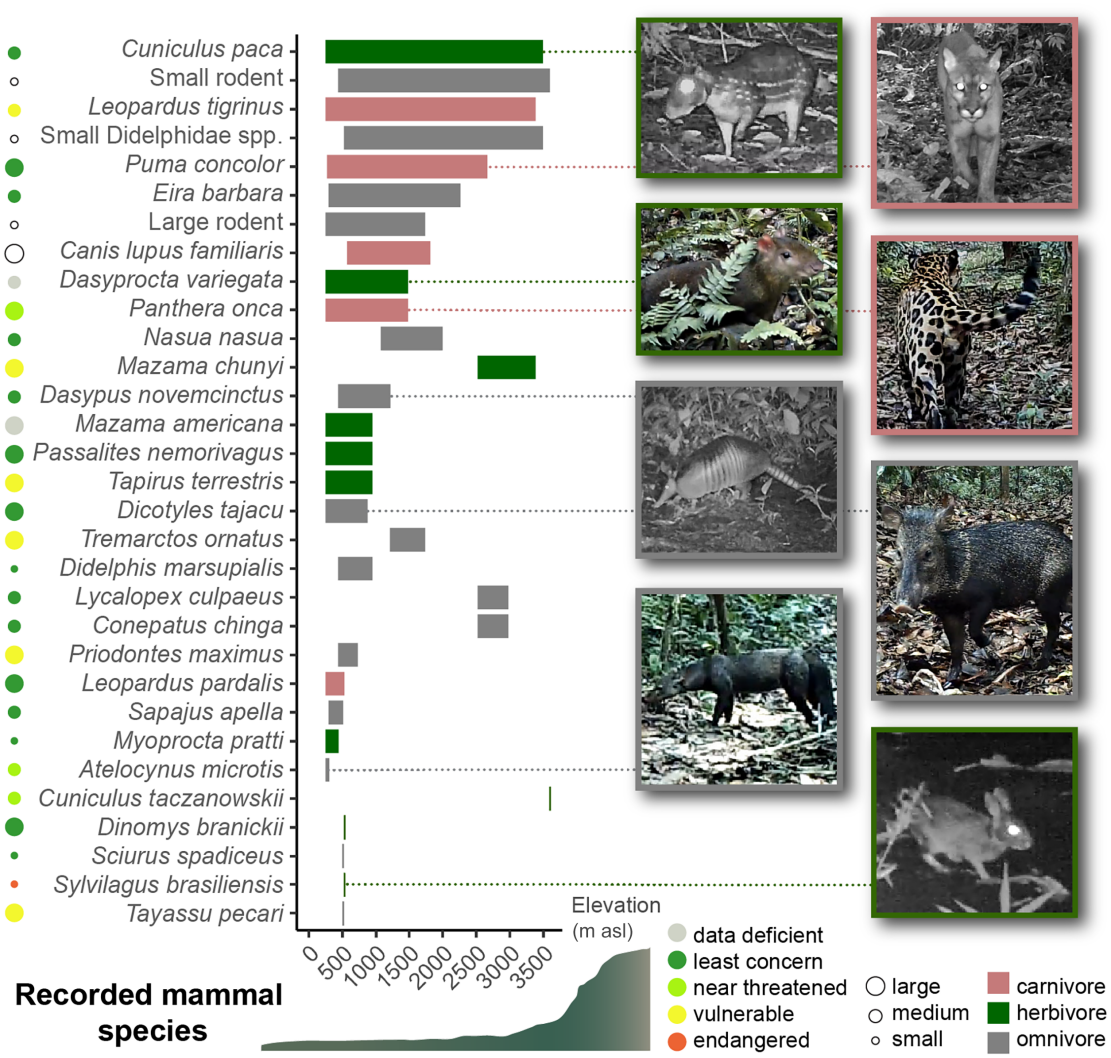
All recorded mammal species and morphospecies (spp.), classified into the trophic groups omnivores, herbivores, and carnivores, and in three different size categories (see dots). IUCN red list status is indicated by the color of the dots. Species list in Appendix S2: Table S2. Photographs by Kim L. Holzmann (screenshots from camera trap videos).

**FIGURE 3:**
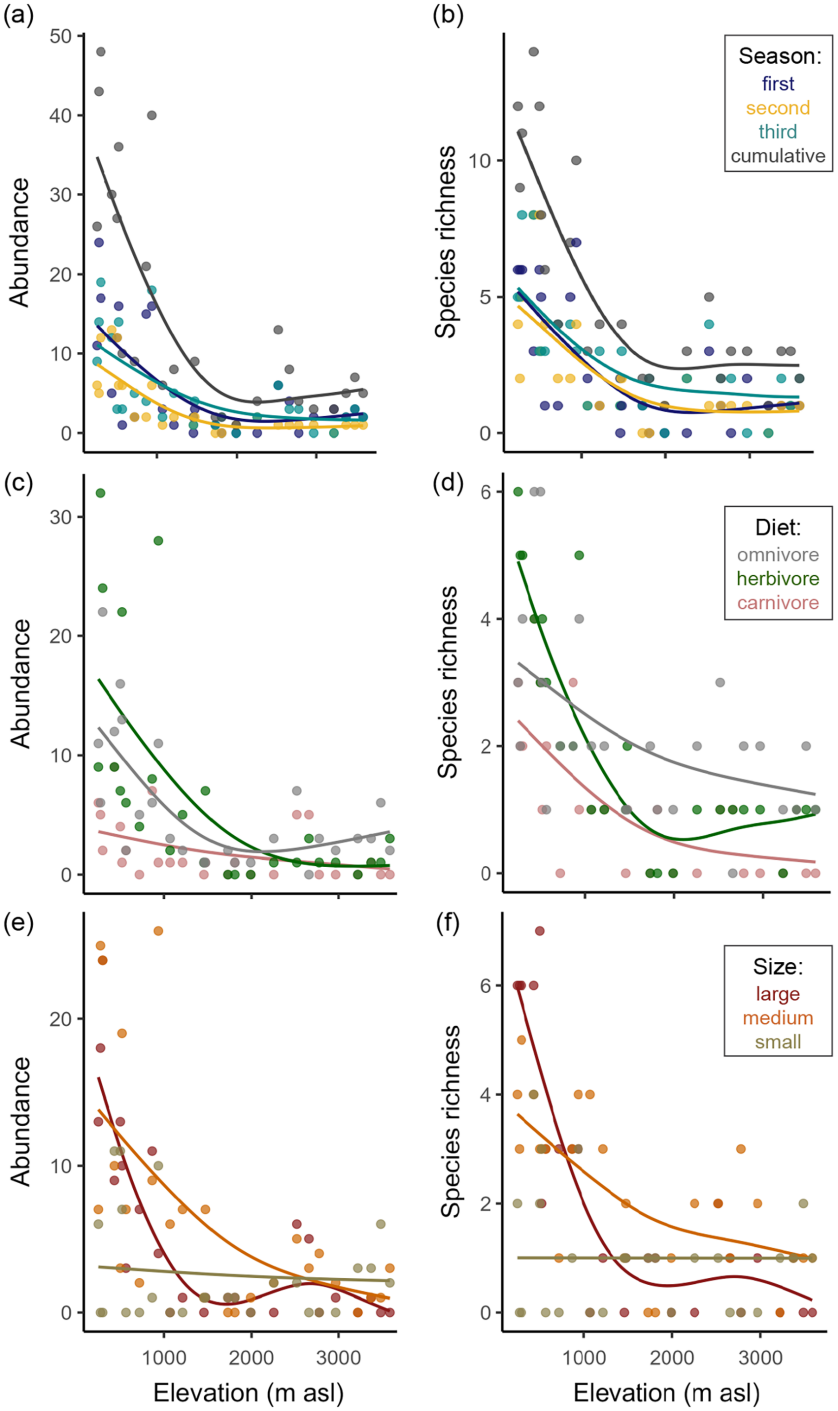
(a) Mammal abundance and (b) species richness patterns across the three sample seasons (Appendix S2: Table S3); first: September–December 2022, second: April–August 2023, third: September–December 2023. (c) Mammal abundance across trophic groups: in the lowlands, herbivores were the most abundant and diverse mammals. Omnivores showed a slight increase in abundance at highest elevations. (d) Species richness patterns across trophic groups: Herbivores showed a slight increase in richness at high elevations (Appendix S2: Table S3). (e) Mammal abundance and (f) species richness patterns across size groups: large mammals decreased exponentially with elevation but showed a smaller peak around 2500 m above sea level. Medium mammals decreased exponentially. Small mammals showed no pattern (Appendix S2: Table S4). Abundances are number of individuals and species richness is observed number of different species. Trend lines are from generalized additive models.

In total, 31 mammal species were recorded across 26 study plots and 52,416 camera trap hours (Figure 2). Twenty‐eight of the species could be identified to species level, while three small rodents were classified as morphospecies. With one exception (domestic dog, recorded twice), all recorded animals were wild mammals. All three trophic groups (carnivores, herbivores and omnivores) were recorded across the whole gradient (Figure 3). The most common species was the lowland paca, *Cuniculus paca* (78 records), followed by the brown agouti, *Dasyprocta variegata* (43 records) and the collared peccary, *Dicotyles tajacu* (35 records). According to IUCN, 16 (55%) of the recorded species were classified as “least concern,” 3 (10%) as “near threatened,” 6 (21%) as “vulnerable” and one (common tapeti, *Sylvilagus brasiliensis*) as “endangered.” A list of all recorded species, their number of records, IUCN status, as well as their trophic and size group, is in the supplement (Appendix S2: Table S2).

### Mammal distribution patterns

Total mammal abundance and species richness decreased significantly with elevation in all field seasons (Figure 3; Appendix S2: Table S3). Species richness also decreased when pooled for 250‐m elevational bands (Appendix S2: Figure S1). Abundance and species richness were highly correlated (*n* = 26, *r* = 0.87, *p* < 0.001). The observed (cumulative) species richness was highly and linearly correlated with the Chao 1 estimated asymptotic species richness (*n* = 26, *r* = 0.95, *p* < 0.001; Appendix S2: Figure S2).

Across trophic groups, most recorded mammal species were omnivores (*n* = 16), followed by herbivores (*n* = 10) and few species classified as carnivores (*n* = 5). Abundance and species richness declined with elevation for all groups (Appendix S2: Table S4). Overall, mean community biomass decreased significantly and exponentially with elevation (gam, effective df = 7.67, residual df = 17.33, *F* = 11.81, *p* < 0.001, deviance explained = 52.70%, *r* ^2^ = 0.49) with similar patterns in carnivores, herbivores, and omnivores (Appendix S2: Figure S3 and Table S4c).

Size classes were represented with 13 large mammal species, 11 medium, and 7 small species. For small mammals, abundances were similarly high across elevations, while medium and large mammals showed strong declines in abundance with elevation (Figure 3). For the species richness of small mammals, no significant trend could be observed; for medium and large mammals, the pattern matched the abundance patterns (Appendix S2: Table S5).

### Measures of NPP as predictors of species richness

**FIGURE 4:**
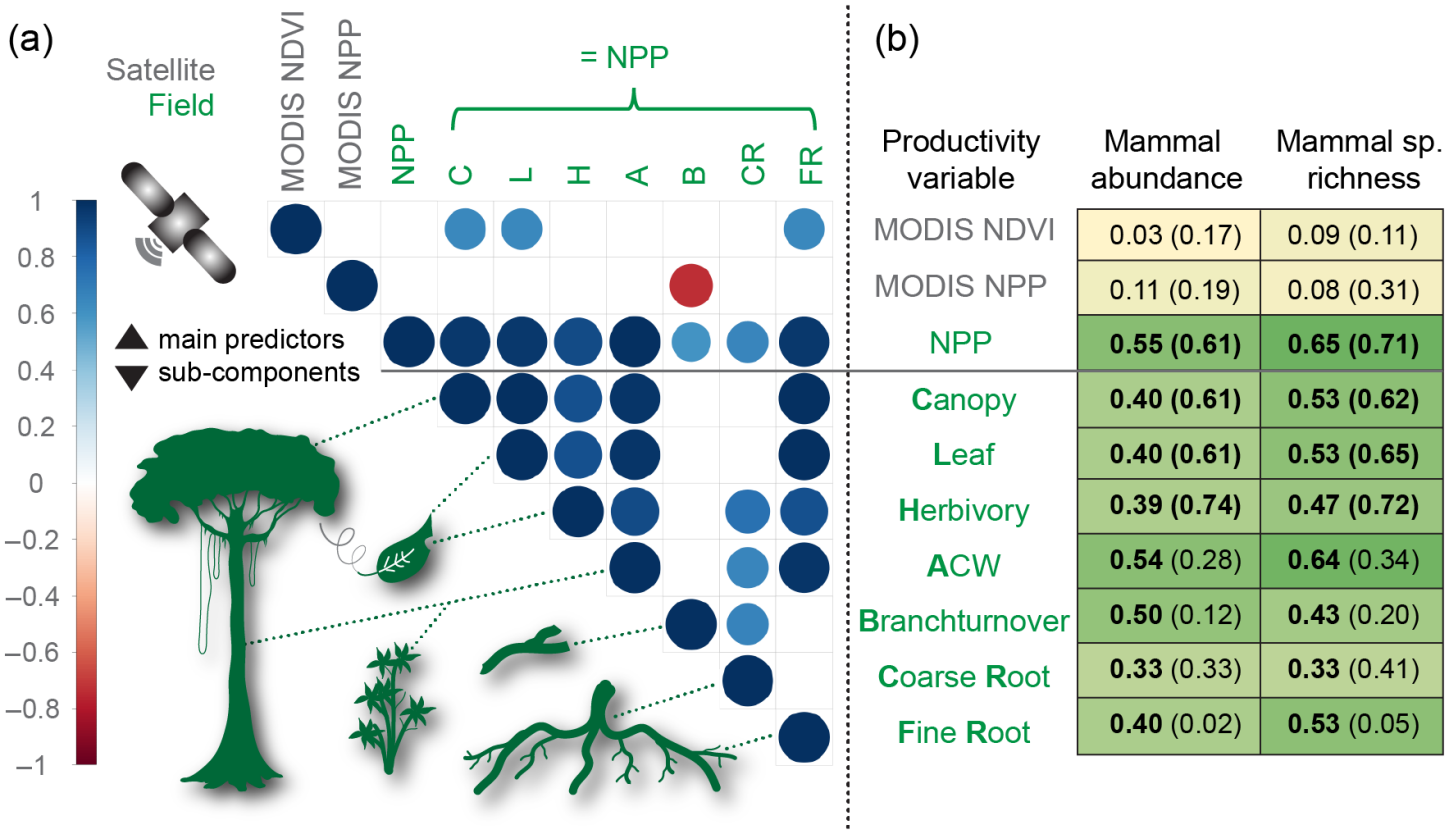
(a) Correlation plot of the various productivity variables (listed in b); the remote‐sensing‐based estimators Normalized Difference Vegetation Index (MODIS NDVI) and net primary productivity (MODIS NPP) and the field‐measured net primary productivity (NPP) measures and its subcomponents (see Malhi et al., 2017). Blank cells in the correlation plot indicate correlations that were not significant at *p* < 0.05. (b) Multiple *r* ^2^ values from single‐factor linear‐regression models (test statistics in Appendix S2: Tables S6 and S7) for the NPP variables and log‐transformed measures of mammal abundance and species richness. In parentheses, values for the reduced dataset (*n* = 9, only plots with original NPP values). Values in bold were significant at *p* < 0.05. Illustrations created by Kim L. Holzmann.

Total field‐measured NPP was not significantly related to its remote‐sensing‐derived proxies MODIS NPP and MODIS NDVI (Figure 4a). While NPP showed a negative exponential decline with increasing elevation, MODIS NPP resulted in a unimodal pattern with a plateau between 1000 and 3000 m asl, and NDVI decreased almost linearly (Appendix S1: Figure S2).

In single‐factor analyses, NPP, MODIS NPP, and MODIS NDVI were compared in their predictive power to explain mammal abundance and richness along the gradient (Appendix S2: Figure S4). Species richness and abundance were significantly correlated to field‐measured NPP, but not to MODIS NPP and MODIS NDVI (Figure 4).

Field‐measured NPP had a significant positive correlation with all its measured components: canopy, leaf, herbivory, ACW, branch turnover, coarse root, and fine root (Figure 4a). MODIS NPP was negatively correlated to field‐measured branch turnover, and MODIS NDVI was positively correlated to canopy, leaf, and fine root values (Figure 4a). For both mammal abundance and species richness, the component ACW showed the strongest correlation, and coarse root the weakest (Figure 4b). All statistical results of single‐factor analyses of NPP, MODIS NPP, and MODIS NDVI are in Appendix S2: Table S6 for abundance and Appendix S2: Table S5 for species richness.

### Direct and indirect effects of climate, energy, and abundance on species richness

Besides NPP, climatic variables were investigated in their predictive power to explain mammal distribution in multifactor models. The best model, that is, with the lowest AIC_c_ value (*F*_2,23_ = 18.92, AIC_c_ = 62.29; all models in Appendix S2: Table S8), explained 62% of the variation in mammal abundance and included NPP as a significant predictor (*p* < 0.001) and MAP (*p* = 0.052). For species richness, the best model (*F*_2,23_ = 26.54, AIC_c_ = 36.96, *R* ^2^ = 0.70; Appendix S2: Tables S9 and S10 with Chao 1 estimator) included the same variables, that is, NPP (*p* < 0.001) and MAP (*p* = 0.071) as predictors of mammalian species richness. For the residuals of the best models, no significant spatial autocorrelation (Moran's *I*) was found (Appendix S2: Table S1).

**FIGURE 5:**
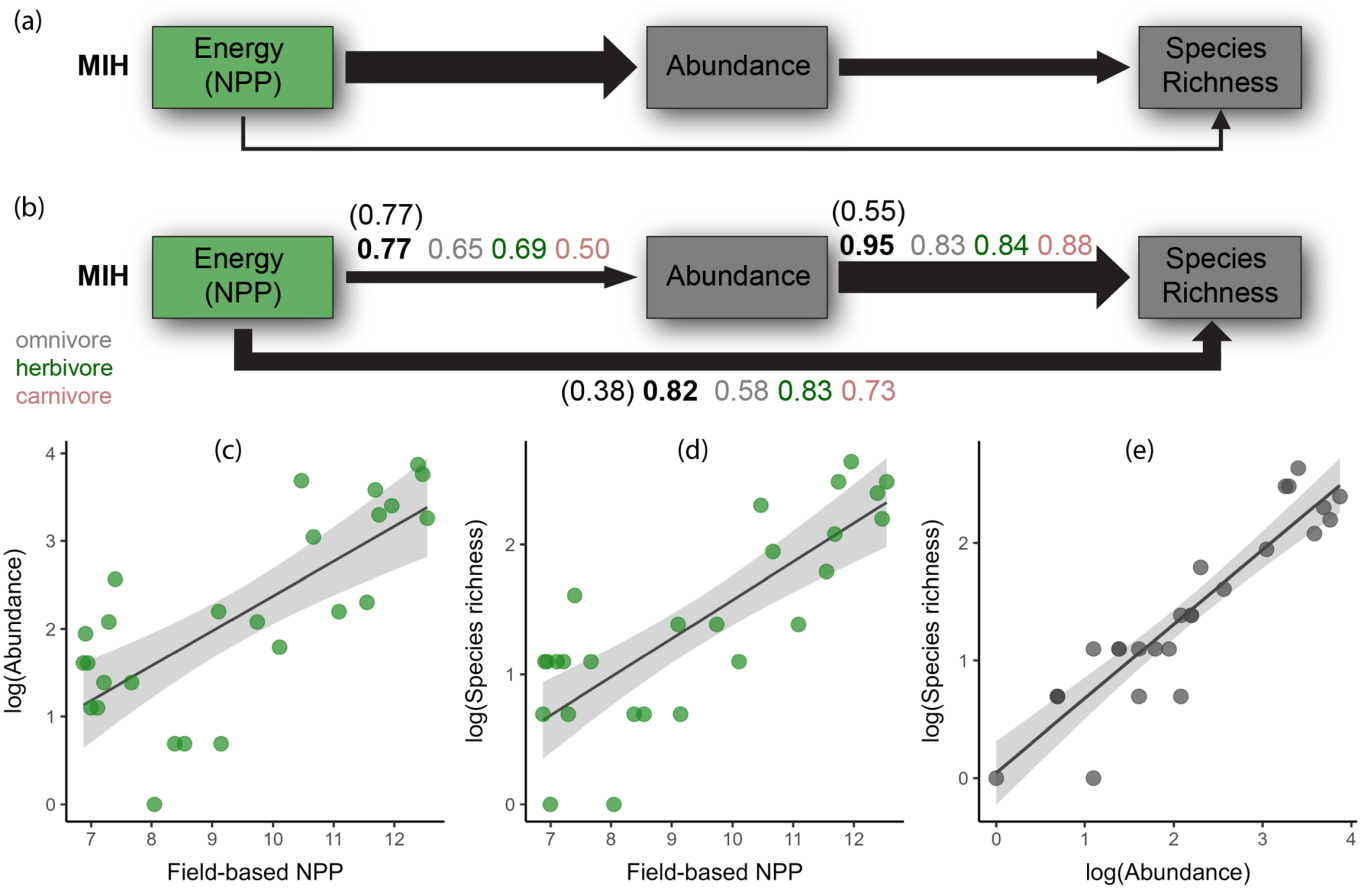
(a) Concept of the more‐individuals hypothesis (MIH). (b) Correlations between available energy (field‐measured net primary productivity, NPP), number of individual mammals (abundance) and mammal species richness were tested for the multitrophic community (numbers in parentheses are standardized beta coefficients, black bold numbers are Pearson's correlation coefficients *r* for comparison with Currie et al., 2004; *n* = 26) and for the separate trophic groups (*r*). For the reduced dataset (only plots with original NPP measurements, *n* = 9) *r* values are: NPP ~ Abundance: 0.78, Abundance ~ Species Richness: 0.92, and NPP ~ Species Richness: 0.84. (c) Linear correlations between mammal abundance and NPP, (d) species richness and NPP (e) species richness and abundance. Gray areas indicate 95% CIs.

Testing the predictions of the MIH (Figure 5a), the correlation species richness–abundance was stronger than the correlation species richness–NPP and the correlation abundance–NPP, both for all mammals and for carnivores and herbivores. For omnivores, abundance was more strongly correlated to NPP than species richness was to NPP, with species richness and abundance again being strongly correlated (Figure 5b–e). Results were the same for the reduced dataset containing only plots with originally measured NPP values (see all correlation coefficients in Figure 5).

## DISCUSSION

In this study, mammal species richness and abundance declined in a strong exponential pattern with elevation from the lowland Amazonian rainforest to the Andean tree line. Trends in species richness and abundance followed those of NPP. Predictions of the MIH were largely fulfilled, suggesting that energy availability limits the number of coexisting mammal species in the Amazonian‐Andean forest mountain ecosystems. Two commonly used remote‐sensing surrogates for NPP (MODIS NPP and MODIS NDVI) were not correlated to field measures of NPP and failed to explain trends in mammal diversity and abundance.

The negative exponential pattern of mammal species richness and abundance does not match the typical unimodal distribution as globally observed in studies focusing exclusively on small mammals (McCain, 2005; McCain et al., 2018). Studies on medium‐ and large‐sized mammals are rare, but large mammals in a study on Mt. Kilimanjaro also displayed a unimodal species richness pattern (Gebert et al., 2019). However, in the Mt. Kilimanjaro study, human impact on mammals in lowland areas might have depressed diversity numbers, since in protected natural habitats mammal diversity was shown to be high in the lowlands as well. Our study included habitat generally undisturbed by humans, with large and intact Amazonian rainforests in the lowlands, potentially allowing the peak of mammal diversity at the lowest elevations. While other studies within the Neotropics generally assessed smaller gradients not reaching the lowlands or higher elevations, they still found similar patterns: species richness of medium to large‐sized mammals was also significantly higher in lower than in higher elevation habitats in Brazil (Pinho et al., 2017), and in the northern Andes of Peru, taxonomic and functional diversity decreased with elevation (Mena & Pacheco, 2022).

### The more‐individual hypothesis revisited—And (largely) confirmed

The most proposed hypothesis to explain biodiversity distributions in the light of climate and energy is the MIH (Figure 5a). One major problem of testing the MIH is to accurately measure energy availability, that is, NPP (Šímová & Storch, 2017; Storch et al., 2018). The detailed NPP measurements in our study allowed a conservative test of the MIH predictions with high‐quality data on energy at the base of the food web: higher energy availability increases the number of individuals within a community and thus species richness through an increasing number of larger and thus more viable populations. In other words, we would expect (1) a strong relationship between abundance and NPP, (2) between species richness and abundance, and (3) between species richness and NPP. In a strict sense, (4) the relationship between NPP and abundance should be stronger than that between NPP and species richness, the latter two being only indirectly linked (Currie et al., 2004).

In our study, expectations (1–3) were clearly fulfilled: mammal abundance and species richness were strongly correlated, confirming the expected pattern following the MIH. Studies on insects (Beck et al., 2011; Kaspari et al., 2000) and birds (Mönkkönen et al., 2006; Pautasso & Gaston, 2005) likewise accepted the MIH after testing and complying with the predictions (1–3). Prediction (4) was rejected in our study: the correlation between NPP and abundance was not stronger than that between NPP and species richness; the strongest correlation in our study was between abundance and species richness. The slightly weaker correlation between abundance and energy in our study might have resulted from the generally very low NPP values in the highlands, where the area bordered the tree line and transitioned into grassland habitat. This might force mammals to be more mobile, which in turn potentially leads to a greater error in the captured abundance values compared with the highly productive lowlands. On the other hand, lower temperatures in the highlands might also constrain the activity hours of mammals. However, all correlations between these three variables were very strong (*r* > 0.77) and the differences were only marginal.

For birds in North America and trees in the Neotropics, the first three predictions were also met, but not the fourth, which led Currie et al. (2004) to reject the MIH in its standard form in their conclusion. Universally, energy–species richness correlations over large spatial gradients seem to be strong (Francis & Currie, 2003; Hawkins et al., 2003; Wright et al., 1993), while energy abundance correlations are often much weaker, as in Currie et al.'s tree data (*r* = 0.70 vs. 0.35; in our study *r* = 0.82 vs. 0.77). The weaker correlation in their study might be associated with the lack of systematic variation in tree abundance through the tropics and having only a few points outside the tropics, as the authors pointed out. Spatially balanced data might lead to a stronger relationship, as in our mammal data sampled from a continuous gradient. Furthermore, they estimated productivity from actual evapotranspiration. If we had used one of our investigated productivity proxies (MODIS NPP/NDVI) instead of the field‐measured NPP, our energy–species richness correlation values would have been similar to the Currie et al. (2004) study (Figure 4b).

Since the MIH assumes interspecific competition for available energy within a community, we additionally separated the multitrophic community into trophic groups (Storch et al., 2018). The predictions (1–3) could also be confirmed for each trophic level separately. Prediction (4) was rejected for herbivores and carnivores, the same pattern than observed for the multitrophic community, but for omnivores (4) was true: Energy–abundance was stronger than energy–species richness, although all correlations were generally high. With this pattern, the omnivorous mammal species fulfill the strict formulation of the MIH; their abundance might be directly driven by available energy, which in turn leads to an increase in species richness. At lower trophic levels, the abundance of smaller mammals might be underestimated due to predation effects or camera trap detection, leading to a weaker correlation with NPP than expected from the MIH. Furthermore, elevation‐dependent predation rates might result in larger underestimation of abundances at low compared with high elevations.

### Field‐measured NPP, but not remotely sensed proxies, predict species richness

Although primary productivity of an ecosystem is known to be a key driver for most ecological processes and patterns, there is no proper consensus on how to measure or estimate it (Šímová & Storch, 2017). One method is to rely on remote‐sensing monitoring, an advancement that made detailed, global data on the Earth's surface easily available. The NDVI is commonly used as a productivity proxy, but with highly variable estimates depending on the sensors and products (Detsch et al., 2016). Particularly in sparsely and densely vegetated areas, NDVI has been shown to underestimate productivity due to backscatter effects and saturation, without accounting for primary production below the canopy cover (Phillips et al., 2008). Moderate Resolution Imaging Spectroradiometer (MODIS) products include further productivity estimators based on more complex algorithms, such as NPP, but only annual values on a spatial scale of 500 m are available. MODIS NPP was a better predictor of bird richness than MODIS NDVI in a landscape with mainly open vegetation (Phillips et al., 2008). A study of small mammals using NDVI and NPP estimates from China found NDVI to be a more suitable proxy for vegetation structure in a subtropical forest, and also concluded that the two measures are not interchangeable (Sun et al., 2020). One of the only other studies that we are aware of, including both field measurements and satellite data, analyzed species richness patterns of small mammals in the Colorado Rocky Mountains investigating NPP from PRISM data (Northwest Alliance for Computational Science and Engineering, https://prism.oregonstate.edu/) and field‐based understory plant biomass as potential predictors (McCain et al., 2018). NPP was identified as the direct explanator for species richness; however, the study did not aim at comparing the performance of field‐ and satellite‐based measurements since understory biomass was measured as a proxy for food availability. More studies comparing complete field measurements of NPP with remote‐sensing estimators may shine light on the actual performance of these proxies and could also help to improve the underlying algorithms.

The detailed field assessment of NPP in our study allowed the investigation of various components of NPP as potential predictors of mammal species richness. We found that total NPP was a better predictor of mammal species richness than any subcomponent of NPP (canopy, leaf, herbivory, ACW, coarse root, fine root), which fits with the idea that trophically complex species assemblages are directly or indirectly (via trophic cascades) dependent on many different rather than on single types of energy resources produced by plants (e.g., fruits, leaves, wood or energy transferred by insect herbivores).

### Conclusions

Our study identified field‐measured NPP—but not remotely sensed proxies—as a strong environmental driver of multitrophic mammal community richness along a large elevational gradient. Evidence is mostly in line with the MIH, suggesting that with an increase in available energy, more individuals and thus a greater number of species can thrive in a region. Remote‐sensing products, such as MODIS NPP and MODIS NDVI, can be useful proxies to explain biodiversity patterns (Gebert et al., 2019), but should be interpreted carefully since they might not capture the actual energy production of ecosystems in highly complex vegetation such as tropical mountain forests (Šímová & Storch, 2017). We want to highlight the importance of cross‐disciplinary work since only the detailed data produced by the vegetation ecologists of the ABERG project allowed deriving the clear relationships between energy and animal species diversity.

Growing anthropogenic impact in the Amazon system, such as land use, logging, and mining, climate change, and concomitant increased fire frequency, drought, and erosion, destroys forest vegetation (Flores et al., 2024), temporarily lowering NPP. Due to the strong relationship between mammals and NPP shown here and in other studies (Gebert et al., 2019; McCain et al., 2018), climate‐ and land‐use‐driven reductions in NPP could potentially diminish mammal abundance and species richness. It is important to monitor and minimize changes in the Amazonian‐Andean forests and other tropical habitats to conserve the unique diversity of mammals depending on these systems.

## AUTHOR CONTRIBUTIONS

Marcell K. Peters, Ingolf Steffan‐Dewenter, Alexander Keller, Gunnar Brehm conceived the idea for the study. Marcell K. Peters designed the study. Kim L. Holzmann collected the data. Kim L. Holzmann analyzed the data with advice from Marcell K. Peters. Kim L. Holzmann wrote the first draft of the manuscript. All authors contributed to the final version of the manuscript.

## CONFLICT OF INTEREST STATEMENT

The authors declare no conflicts of interest.

## Supporting information


Appendix S1:

Appendix S2:

## Data Availability

Data (Holzmann, 2025) are available in Zenodo at https://doi.org/10.5281/zenodo.14746131.
