# Supplementary material for: Net primary productivity but not its remote‐sensing proxies predict mammal diversity in Andean‐Amazonian rainforests
Source: Ecology. 2025 Mar 10;106(3):e70059. doi: 10.1002/ecy.70059 (PMC11894361; doi:10.1002/ecy.70059)
Supplement: Supplementary file 1 — Appendix S1: [file ECY-106-e70059-s001.pdf]

Ecology

Appendix S1 for:

Net primary productivity but not its remote sensing proxies predict mammal diversity in Andean-Amazonian rainforests

Kim L. Holzmann, Pedro Alonso-Alonso, Yenny Correa-Carmona, Andrea Pinos, Felipe Yon, Alejandro Lopera, Gunnar Brehm, Alexander Keller, Ingolf Steffan-Dewenter, Marcell K. Peters

Environmental data

Mean annual precipitation (MAP) data was available from the long-term research of the ABERG project (Andes Biodiversity and Ecosystem Research Group). We used the measurements from Huaraca Huasco et al. (2021) collected from automatic weather stations, if available for the exact same study plot (N = 7), or clustered for plots at similar elevations (N = 19; mean elevational difference = 103 m).

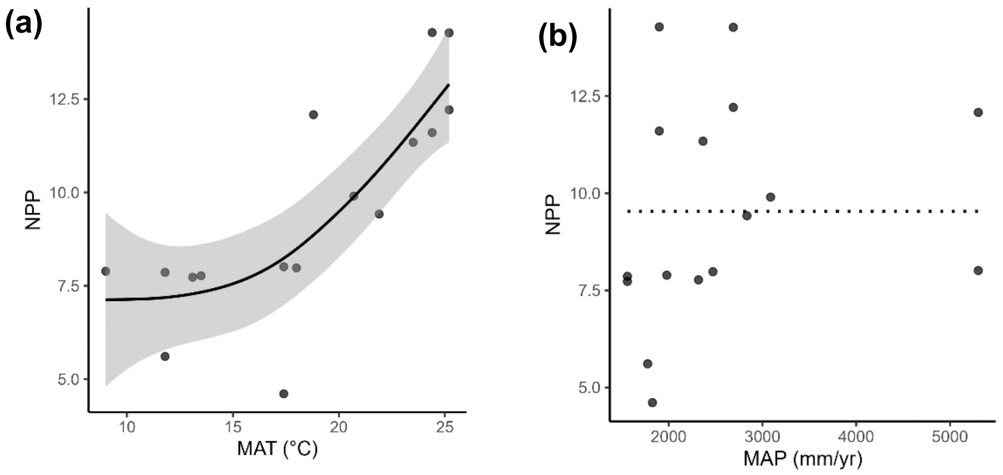

Figure S1: Relations between field-measured net primary productivity (NPP) and (a) mean annual temperature (MAT) and (b) mean annual precipitation (MAP). Trend lines are gam functions. Dotted line represents a non-significant relation. Grey area indicates 95% confidence interval.

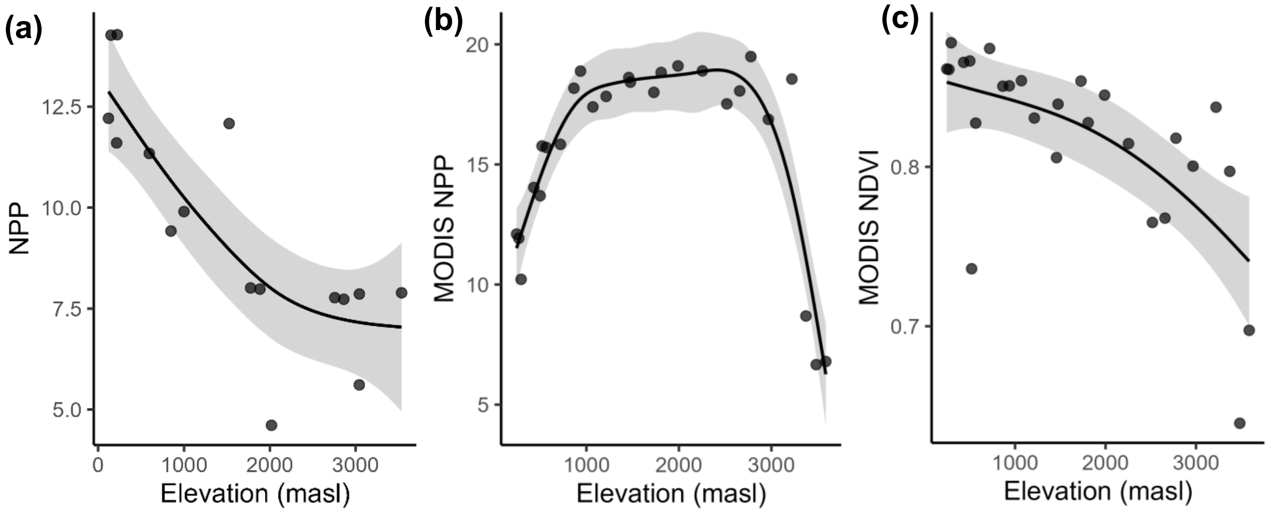

Figure S2: Elevational trends of (a) field-measured, total net primary productivity (NPP) data from ABERG (Malhi et al., 2017), (b) remote sensing-based MODIS NPP and (c) MODIS Normalized Difference Vegetation Index (NDVI). Grey area indicates 95% confidence interval.
