# Supplementary material for: Net primary productivity but not its remote‐sensing proxies predict mammal diversity in Andean‐Amazonian rainforests
Source: Ecology. 2025 Mar 10;106(3):e70059. doi: 10.1002/ecy.70059 (PMC11894361; doi:10.1002/ecy.70059)
Supplement: Supplementary file 2 — Appendix S2: [file ECY-106-e70059-s002.pdf]

Ecology

Appendix S2 for:

Net primary productivity but not its remote sensing proxies predict mammal diversity in Andean-Amazonian rainforests

Kim L. Holzmann, Pedro Alonso-Alonso, Yenny Correa-Carmona, Andrea Pinos, Felipe Yon, Alejandro Lopera, Gunnar Brehm, Alexander Keller, Ingolf Steffan-Dewenter, Marcell K. Peters

Alternative mammal analyses

Linear models to investigate predictors for mammal abundance and species richness were also conducted with a reduced dataset containing only plots with original, in the field-measured net primary productivity (NPP) values. Results generally led to the same conclusions (Appendix S2 Table S6, Table S7).

In an alternative analysis using the Chao 1 estimated species richness instead of the observed number of species as the response variable and mean annual temperature (MAT), mean annual precipitation (MAP) and NPP as explanatory variables, the best model (AICc = 47.05, R^2 = 0.59) included NPP as the only explanatory variable (Appendix S2 Table S10).

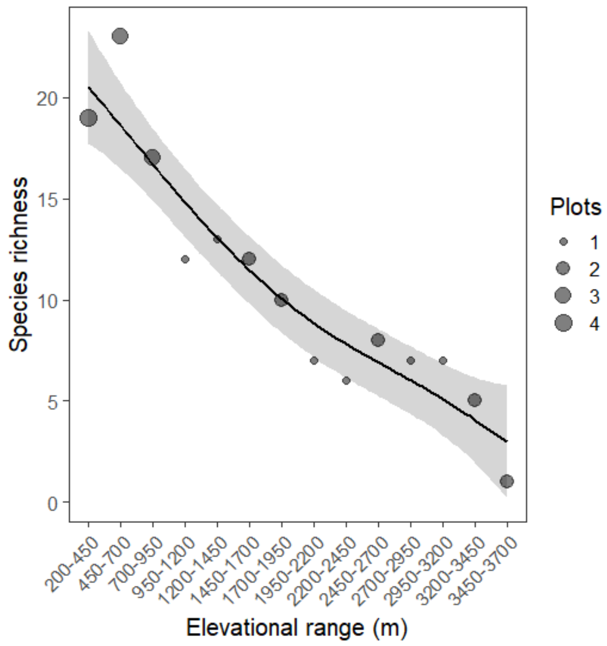

Figure S1: Mammalian species richness pooled for 250-m elevational bands. Circle size represents the number of plots included in the specific range. Grey area indicates 95% confidence interval.

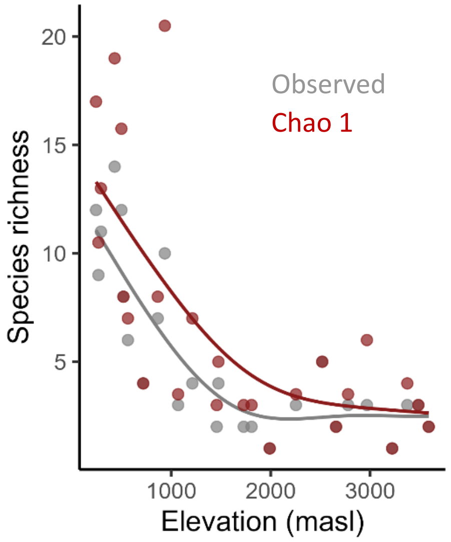

Figure S2: Observed and Chao 1 estimated mammal species richness across the elevational gradient. Both indices were highly correlated (Pearson's correlation test, n = 26, r = 0.95, p < 0.001).

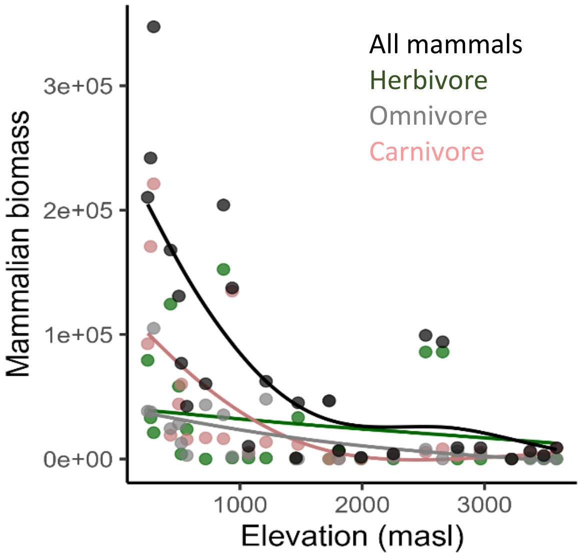

Figure S3: Mammalian biomass for each study plot (means from the three field seasons) across the elevational gradient for the multitrophic community (black) and the three different trophic groups (test statistics in Appendix S2: Table S4c).

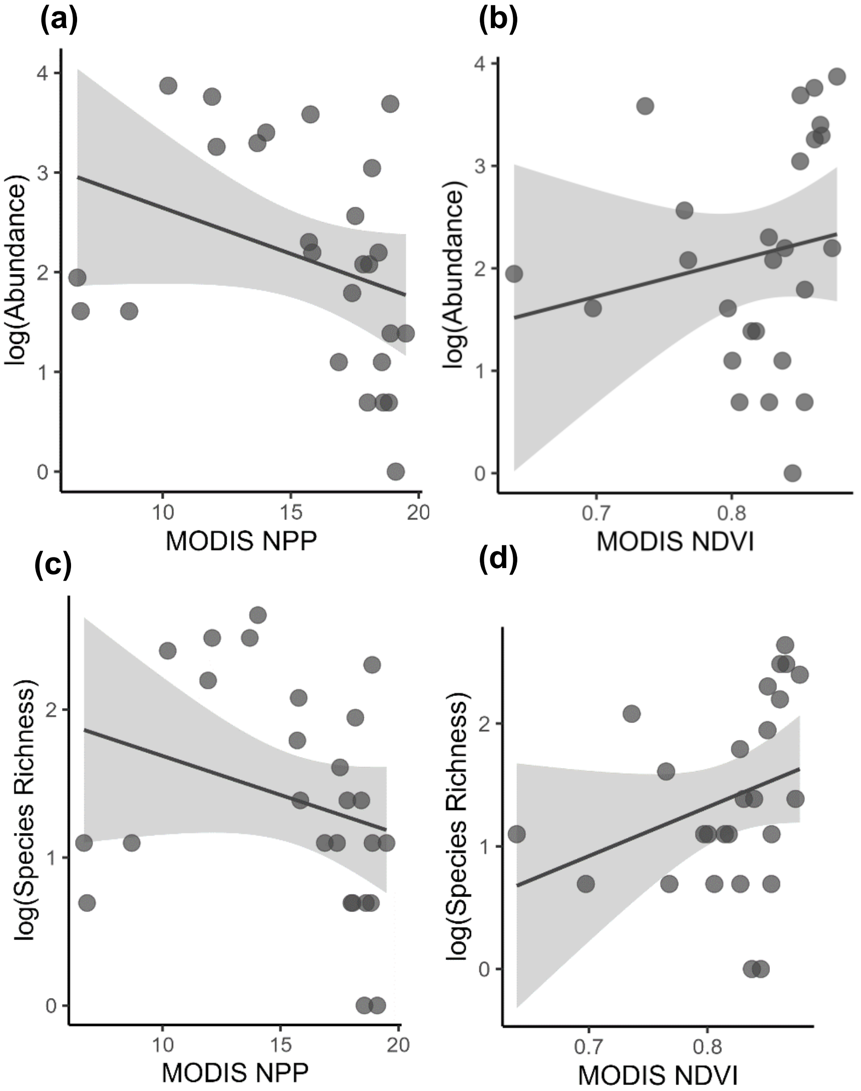

Figure S4: Linear correlations between (a) mammal abundance and remote sensing-based estimates of net primary productivity (MODIS NPP), (b) abundance and MODIS Normalized Difference Vegetation Index (NDVI), (c) species richness and MODIS NPP, (d) species richness and MODIS NDVI. Grey area indicates 95% confidence interval.

Table S1: Spatial autocorrelation tests using Moran's I of (a) residuals of generalized additive models on mammal abundance and (b) mammal species richness across the three field seasons (Season 1: September-December 2022, Season 2: April- August 2023, Season 3: September- December 2023), and (c) of residuals from the identified best linear models to explain mammal abundance and species richness. Expected Moran's I under the null hypothesis of no spatial autocorrelation was E[I] = -1/(26-1) = -0.04 (Moran, 1948).

**(a)**	Term	Moran's I observed	p value
Season 1	s(Elevation)	0.089	0.254
Season 2	s(Elevation)	0.004	0.699
Season 3	s(Elevation)	-0.046	0.961

**(b)**	Term	Moran's I observed	p value
Season 1	s(Elevation)	0.137	0.117
Season 2	s(Elevation)	0.152	0.087
Season 3	s(Elevation)	-0.219	0.117

**(c)**	Moran's I observed	p value
Abundance ~ MAP + NPP	0.120	0.161
Species richness ~ MAP + NPP	0.092	0.249

Table S2: List of all recorded mammal species and morphospecies (*spp.*) with their elevational occurrence, their classification into a trophic guild and size group as in Bogoni et al. (2016), their average body mass in g from Wilman et al. (2014), and their IUCN Red List status (lc, least concern; vu, vulnerable; nt, near threatened; en, endangered; dd, data deficient). Order according to range size as in Figure 2. Range = Elevational range size (maximum-minimum observed elevation), min = lowest elevation at which a species was observed, max = highest elevation at which at species was observed. Records are the number of total observations of the species, considering the hourly event count (Hegerl et al., 2017). Some rodents that could not be distinguished from camera trap videos were classified as a small and large morphospecies.

Species	Records	min	max	Range	Trophic guild	Size	Mass (g)	IUCN
Cuniculus paca	78	245	3484	3239	herbivore	medium	8172.55	lc
Small rodent	27	432	3588	3156	omnivore	small	16.25	-
Leopardus tigrinus	9	245	3373	3128	carnivore	medium	2250.00	vu
Small Didelphidae spp	8	520	3484	2964	omnivore	small	41.48	-
Puma concolor	20	269	2657	2388	carnivore	large	51600.04	lc
Eira barbara	5	294	2254	1960	omnivore	medium	3910.03	lc
Large rodent	20	245	1729	1484	omnivore	small	229.13	-
Canis lupus familiaris	2	565	1808	1243	carnivore	large	20000.00	-
Dasyprocta variegata	43	245	1475	1230	herbivore	medium	2674.98	dd
Panthera onca	9	245	1475	1230	carnivore	large	100000.00	nt
Nasua nasua	2	1070	1990	920	omnivore	medium	3793.85	lc
Mazama chunyi	3	2518	3373	855	herbivore	large	16499.85	vu
Dasypus novemcinctus	17	432	1213	781	omnivore	medium	4203.78	lc
Mazama americana	21	245	936	691	herbivore	large	22799.75	dd
Passalites nemorivagus	8	245	936	691	herbivore	large	16633.17	lc
Tapirus terrestris	5	245	936	691	herbivore	large	207500.91	vu
Dicotyles tajacu	35	245	865	620	omnivore	large	21266.69	lc

Tremarctos ornatus	2	1213	1729	516	omnivore	large	140000.6	vu
Didelphis marsupialis	2	432	936	504	omnivore	small	1091.16	lc
Lycalopex culpaeus	5	2518	2967	449	omnivore	medium	8616.24	lc
Conepatus chinga	5	2518	2967	449	omnivore	medium	2085.02	lc
Priodontes maximus	2	432	719	287	omnivore	large	45359.68	vu
Leopardus pardalis	12	245	520	275	carnivore	large	11900.08	lc
Sapajus apella	4	294	500	206	omnivore	medium	2500.00	lc
Myoprocta pratti	3	245	432	187	herbivore	small	966.73	lc
Atelocynus microtis	4	245	294	49	omnivore	medium	7749.97	nt
Cuniculus taczanowskii	3	3588	3588	0	herbivore	medium	8999.95	nt
Dinomys branickii	9	520	520	0	herbivore	large	12500.00	lc
Sciurus spadiceus	5	500	500	0	omnivore	small	403.33	lc
Sylvilagus brasiliensis	3	520	520	0	herbivore	small	949.99	en
Tayassu pecari	1	500	500	0	omnivore	large	32233.69	vu

Table S3: Effect of elevation in generalized additive models on (a) mammal abundance and (b) mammal species richness across the three field seasons. Season 1: September-December 2022, Season 2: April-August 2023, Season 3: September-December 2023. edf = effective degrees of freedom, Res. df = residual degrees of freedom, Dev. Expl. = deviance explained. Refers to Figure 3a, b.

**(a)**	Term	edf	Res. df	F value	p value	Dev. Expl. (%)
Cumulative	s(Elevation)	2.511	22.489	14.651	<0.001	68.04
Season 1	s(Elevation)	2.222	22.778	8.428	0.001	51.57
Season 2	s(Elevation)	2.528	22.472	13.562	<0.001	66.54
Season 3	s(Elevation)	2.022	22.978	8.316	0.001	48.593

**(b)**	Term	edf	Res. df	F value	p value	Dev. Expl. (%)
Cumulative	s(Elevation)	2.695	22.305	20.231	<0.001	75.84
Season 1	s(Elevation)	2.307	22.693	10.251	<0.001	57.50
Season 2	s(Elevation)	2.197	22.803	9.926	<0.001	55.19
Season 3	s(Elevation)	2.530	22.470	9.523	<0.001	58.48

Table S4: Effect of elevation in generalized additive models on (a) mammal abundance, (b) mammal species richness and (c) mean biomass for the different trophic guilds. edf = effective degrees of freedom, Res. df = residual degrees of freedom, Dev. Expl. = deviance explained. Refers to Figure 3c, d and Figure S3.

**(a)**	Term	edf	Res. df	F value	p value	Dev. Expl. (%)
Herbivores	s(Elevation)	1.946	23.054	8.777	0.001	49.18
Carnivores	s(Elevation)	2.560	22.440	3.265	0.041	33.83
Omnivores	s(Elevation)	2.389	22.611	8.805	<0.001	54.895

**(b)**	Term	edf	Res. df	F value	p value	Dev. Expl. (%)
Herbivores	s(Elevation)	2.929	22.071	24.004	<0.001	80.35
Carnivores	s(Elevation)	2.027	22.973	11.151	<0.001	55.71
Omnivores	s(Elevation)	1.704	23.296	5.375	0.012	34.16

**(c)**	Term	edf	Res. df	F value	p value	Dev. Expl. (%)
Herbivores	s(Elevation)	-0.001	25.001	-2.338	0.028	18.50
Carnivores	s(Elevation)	-0.003	25.003	-3.390	0.002	32.40
Omnivores	s(Elevation)	-0.002	25.002	-4.297	<0.001	43.50

Table S5: Effect of elevation in generalized additive models on (a) mammal abundance and (b) mammal species richness for the different size classes. Species were classified according to their body size as "small" ≤ 1.2 kg, "medium" > 1.2 and ≤10 kg, and "large" mammals >10 kg (Bogoni et al., 2016). edf = effective degrees of freedom, Res. df = residual degrees of freedom, Dev. Expl. = deviance explained. Refers to Figure 3e,f.

**(a)**	Term	edf	Res. df	F value	p value	Dev. Expl. (%)
Small	s(Elevation)	1.763	23.237	1.512	0.225	15.95
Medium	s(Elevation)	1.737	23.263	7.554	0.003	42.37
Large	s(Elevation)	3.501	21.499	16.200	<0.001	77.33

**(b)**	Term	edf	Res. df	F value	p value	Dev. Expl. (%)
Small	s(Elevation)	1.313	23.687	0.500	0.458	6.87
Medium	s(Elevation)	7.792	17.208	7.401	<0.001	79.80
Large	s(Elevation)	3.239	21.761	24.025	<0.001	82.05

Table S6: Effect of single net primary productivity (NPP) predictor variables on the log-transformed abundance (number of individuals) of mammals for (a) the full dataset and (b) a reduced dataset only including plots with actual NPP data. Shown are results of linear models. Explanatory variables are total NPP and its seven sub-components. In addition, they were compared to satellite-based surrogates of NPP (MODIS NPP) and the normalized difference vegetation index (MODIS NDVI). Num. df = numerator degrees of freedom, Denom. df = denominator degrees of freedom. ACW = aboveground coarse wood.

(a) Full dataset

Variable	Intercept	Slope	R^2	F value	Num. df	Denom. df	P value
NPP_Total	-1.598	0.397	0.553	29.693	1.000	24.000	<0.001
NPP_Canopy	-0.988	0.734	0.404	16.264	1.000	24.000	<0.001
NPP_Leaf	-1.224	1.180	0.404	16.264	1.000	24.000	<0.001
NPP_Herbivory	0.504	3.322	0.395	15.666	1.000	24.000	<0.001
NPP_ACW	-1.703	1.992	0.538	27.974	1.000	24.000	<0.001
NPP_BranchTurnover	-0.933	5.033	0.503	24.292	1.000	24.000	<0.001
NPP_CoarseRoot	0.306	3.857	0.329	11.749	1.000	24.000	0.002
NPP_FineRoot	-0.480	1.234	0.404	16.264	1.000	24.000	<0.001
MODIS NPP	3.569	-0.092	0.108	2.905	1.000	24.000	0.101
MODIS NDVI	-0.669	3.420	0.031	0.779	1.000	24.000	0.386

(b) Reduced dataset

Variable	Intercept	Slope	R^2	F value	Num. df	Denom. df	P value
NPP_Total	-1.742	0.397	0.609	10.886	1.000	7.000	0.013
NPP_Canopy	-1.078	0.689	0.607	10.829	1.000	7.000	0.013
NPP_Leaf	-1.057	1.008	0.609	10.904	1.000	7.000	0.013
NPP_Herbivory	-0.608	5.420	0.743	20.279	1.000	7.000	0.003
NPP_ACW	0.280	0.767	0.277	2.687	1.000	7.000	0.145
NPP_BranchTurnover	0.096	3.048	0.120	0.957	1.000	7.000	0.360
NPP_CoarseRoot	0.288	3.471	0.330	3.441	1.000	7.000	0.106
NPP_FineRoot	1.068	0.267	0.018	0.132	1.000	7.000	0.728
MODIS NPP	7.106	-0.308	0.193	1.676	1.000	7.000	0.237
MODIS NDVI	-20.191	25.981	0.175	1.480	1.000	7.000	0.263

Table S7: Effect of single net primary productivity (NPP) predictor variables on the log-transformed species richness of mammals for (a) the full dataset and (b) a reduced dataset only including plots with actual field NPP data. Shown are results of linear models. Explanatory variables are total NPP and its seven sub-components. In addition, they were compared to satellite-based surrogates of NPP (MODIS NPP) and the normalized difference vegetation index (MODIS NDVI). Num. df = numerator degrees of freedom, Denom. df = denominator degrees of freedom. ACW = aboveground coarse wood.

(a) Full dataset

Variable	Intercept	Slope	R^2	F value	Num. df	Denom. df	P value
NPP_Total	-1.385	0.296	0.650	44.653	1.000	24.000	<0.001
NPP_Canopy	-1.061	0.578	0.530	27.055	1.000	24.000	<0.001
NPP_Leaf	-1.247	0.929	0.530	27.055	1.000	24.000	<0.001
NPP_Herbivory	0.167	2.503	0.475	21.709	1.000	24.000	<0.001
NPP_ACW	-1.476	1.490	0.638	42.339	1.000	24.000	<0.001
NPP_BranchTurnover	-0.544	3.180	0.426	17.785	1.000	24.000	<0.001
NPP_CoarseRoot	0.134	2.659	0.331	11.874	1.000	24.000	0.002
NPP_FineRoot	-0.662	0.971	0.530	27.055	1.000	24.000	<0.001
MODIS NPP	2.215	-0.053	0.075	1.942	1.000	24.000	0.176
MODIS NDVI	-1.869	3.986	0.090	2.387	1.000	24.000	0.135

(b) Reduced dataset

Variable	Estimate	Std. Error	R^2	F value	Num. df	Denom. df	P value
NPP_Total	-1.455	0.304	0.712	17.278	1.000	7.000	0.004
NPP_Canopy	-0.814	0.493	0.621	11.469	1.000	7.000	0.012
NPP_Leaf	-0.842	0.738	0.651	13.061	1.000	7.000	0.009
NPP_Herbivory	-0.439	3.787	0.723	18.282	1.000	7.000	0.004
NPP_ACW	0.073	0.600	0.339	3.584	1.000	7.000	0.100
NPP_BranchTurnover	-0.269	2.794	0.201	1.765	1.000	7.000	0.226
NPP_CoarseRoot	0.065	2.754	0.413	4.933	1.000	7.000	0.062
NPP_FineRoot	0.512	0.303	0.047	0.347	1.000	7.000	0.575
MODIS NPP	6.039	-0.276	0.309	3.124	1.000	7.000	0.120
MODIS NDVI	-10.963	14.382	0.107	0.835	1.000	7.000	0.391

Table S8: Model support for linear models using the log-transformed response variable abundance (number of individuals) of mammals and the explanatory variables mean annual temperature (MAT), mean annual precipitation (MAP), and net primary productivity (NPP; measured in the field or satellite surrogate MODIS NPP) or normalized difference vegetation index (MODIS NDVI) in multiple regression models.

Model comparison: Predictors of abundance

Intercept	MAT	MAP	**Field NPP**	R^2	df	logLik	AICc	delta	weight
-1.246		~-0.001	0.427	0.622	4.000	-26.195	62.294	<0.001	0.304
-3.064	-0.197		0.911	0.621	4.000	-26.218	62.341	0.047	0.297
-2.451	-0.151	~-0.001	0.814	0.659	5.000	-24.871	62.742	0.448	0.243
-1.598			0.397	0.553	3.000	-28.374	63.839	1.545	0.141
0.378	0.145	~-0.001		0.508	4.000	-29.623	69.151	6.857	0.010
-0.056	0.128			0.421	3.000	-31.731	70.553	8.259	0.005
2.128				<0.001	2.000	-38.842	82.206	19.912	<0.001
2.371		~-0.001		0.011	3.000	-38.702	84.495	22.201	<0.001
Intercept	MAT	MAP	**MODIS NPP**	R^2	df	logLik	AICc	delta	weight
1.591	0.138		-0.116	0.591	4.000	-27.235	64.374	<0.001	0.531
1.678	0.148	~-0.001	-0.102	0.628	5.000	-25.979	64.958	0.584	0.396
0.378	0.145	~-0.001		0.508	4.000	-29.623	69.151	4.777	0.049
-0.056	0.128			0.421	3.000	-31.731	70.553	6.179	0.024
3.569			-0.092	0.108	3.000	-37.357	81.805	17.431	<0.001
2.128				<0.001	2.000	-38.842	82.206	17.832	<0.001
2.371		~-0.001		0.011	3.000	-38.702	84.495	20.121	<0.001
3.582		~-0.001	-0.091	0.108	4.000	-37.355	84.615	20.241	<0.001
Intercept	MAT	MAP	**MODIS NDVI**	R^2	df	logLik	AICc	delta	weight
5.221	0.188	~-0.001	-6.961	0.582	5.000	-27.507	68.014	<0.001	0.376
5.581	0.181		-7.991	0.521	4.000	-29.261	68.428	0.414	0.305
0.378	0.145	~-0.001		0.508	4.000	-29.623	69.151	1.137	0.213
-0.056	0.128			0.421	3.000	-31.731	70.553	2.539	0.106
2.128				<0.001	2.000	-38.842	82.206	14.192	<0.001
-0.669			3.420	0.031	3.000	-38.427	83.945	15.931	<0.001
2.371		~-0.001		0.011	3.000	-38.702	84.495	16.481	<0.001
-1.075		~-0.001	4.409	0.058	4.000	-38.061	86.027	18.013	<0.001

Table S9: Model support for linear models using the log-transformed response variable species richness of mammals and the explanatory variables mean annual temperature (MAT), mean annual precipitation (MAP), and net primary productivity (NPP; measured in the field or satellite surrogate MODIS NPP) or normalized difference vegetation index (MODIS NDVI) in multiple regression models. Numbers in the first four columns are parameter estimates of the variable indicated in the header line.

Model selection: Predictors of species richness

Intercept	MAT	MAP	**Field NPP**	R^2	df	logLik	AICc	delta	weight
-1.185		~-0.001	0.313	0.698	4.000	-13.526	36.957	<0.001	0.419
-1.385			0.296	0.650	3.000	-15.415	37.921	0.964	0.259
-1.992	-0.081		0.508	0.675	4.000	-14.457	38.820	1.862	0.165
-1.604	-0.052	~-0.001	0.447	0.707	5.000	-13.118	39.236	2.278	0.134
-0.050	0.110	~-0.001		0.611	4.000	-16.814	43.533	6.576	0.016
-0.313	0.100			0.543	3.000	-18.896	44.882	7.925	0.008
1.390				<0.001	2.000	-29.078	62.678	25.721	<0.001
1.465		~-0.001		0.002	3.000	-29.050	65.192	28.234	<0.001

Intercept	MAT	MAP	**MODIS NPP**	R^2	df	logLik	AICc	delta	weight
0.696	0.106		-0.071	0.678	4.000	-14.361	38.626	<0.001	0.538
0.749	0.112	~-0.001	-0.062	0.707	5.000	-13.130	39.261	0.634	0.392
-0.050	0.110	~-0.001		0.611	4.000	-16.814	43.533	4.907	0.046
-0.313	0.100			0.543	3.000	-18.896	44.882	6.256	0.024
1.390				<0.001	2.000	-29.078	62.678	24.052	<0.001
2.215			-0.053	0.075	3.000	-28.067	63.225	24.598	<0.001
1.465		~-0.001		0.002	3.000	-29.050	65.192	26.566	<0.001
2.189		<0.001	-0.055	0.076	4.000	-28.052	66.010	27.383	<0.001

Intercept	MAT	MAP	**MODIS NDVI**	R^2	df	logLik	AICc	delta	weight
-0.050	0.110	~-0.001		0.611	4.000	-16.814	43.533	<0.001	0.350
2.262	0.131	~-0.001	-3.323	0.646	5.000	-15.564	44.127	0.594	0.260
2.489	0.126		-3.973	0.596	4.000	-17.311	44.527	0.994	0.213
-0.313	0.100			0.543	3.000	-18.896	44.882	1.349	0.178
1.390				<0.001	2.000	-29.078	62.678	19.145	<0.001
-1.869			3.986	0.090	3.000	-27.846	62.782	19.249	<0.001
-2.112		~-0.001	4.577	0.111	4.000	-27.552	65.008	21.475	<0.001
1.465		~-0.001		0.002	3.000	-29.050	65.192	21.659	<0.001

Table S10: Model support for linear models using the log-transformed response variable Chao 1 estimated asymptotic species richness of mammals and the explanatory variables mean annual temperature (MAT), mean annual precipitation (MAP), and net primary productivity (NPP; measured in the field or satellite surrogate MODIS NPP) or normalized difference vegetation index (MODIS NDVI) in multiple regression models. Numbers in the first four columns are parameter estimates of the variable indicated in the header line.

Model selection: Predictors of species richness with Chao 1 estimated asymptotic species richness

Intercept	MAT	MAP	**Field NPP**	R^2	df	logLik	AICc	delta	weight
-1.282			0.308	0.587	3.000	-19.979	47.050	<0.001	0.516
-1.173		~-0.001	0.317	0.599	4.000	-19.606	49.116	2.067	0.184
-1.716	-0.058		0.460	0.597	4.000	-19.643	49.190	2.141	0.177
-0.196	0.106			0.507	3.000	-22.267	51.624	4.575	0.052
-1.522	-0.044	~-0.001	0.430	0.604	5.000	-19.429	51.858	4.808	0.047
-0.029	0.112	~-0.001		0.530	4.000	-21.656	53.216	6.167	0.024
1.609				<0.001	2.000	-31.473	67.468	20.419	<0.001
1.517		<0.001		0.003	3.000	-31.438	69.966	22.917	<0.001

Intercept	MAT	MAP	**MODIS NPP**	R^2	df	logLik	AICc	delta	weight
0.655	0.111		-0.060	0.587	4.000	-19.971	49.847	<0.001	0.538
-0.196	0.106			0.507	3.000	-22.267	51.624	1.778	0.221
0.683	0.114	~-0.001	-0.056	0.594	5.000	-19.769	52.538	2.691	0.140
-0.029	0.112	~-0.001		0.530	4.000	-21.656	53.216	3.370	0.100
1.609				<0.001	2.000	-31.473	67.468	17.622	<0.001
2.246			-0.041	0.037	3.000	-30.981	69.052	19.206	<0.001
1.517		<0.001		0.003	3.000	-31.438	69.966	20.120	<0.001
2.149		<0.001	-0.048	0.050	4.000	-30.813	71.531	21.684	<0.001

Intercept	MAT	MAP	**MODIS NDVI**	R^2	df	logLik	AICc	delta	weight
-0.196	0.106			0.507	3.000	-22.267	51.624	<0.001	0.508
-0.029	0.112	~-0.001		0.530	4.000	-21.656	53.216	1.592	0.229
1.548	0.122		-2.473	0.524	4.000	-21.813	53.530	1.906	0.196
1.404	0.125	~-0.001	-2.060	0.541	5.000	-21.336	55.673	4.048	0.067
-2.675			5.238	0.130	3.000	-29.663	66.417	14.793	<0.001
1.609				<0.001	2.000	-31.473	67.468	15.844	<0.001
-2.783		~-0.001	5.502	0.133	4.000	-29.613	69.131	17.506	<0.001
1.517		<0.001		0.003	3.000	-31.438	69.966	18.342	<0.001

References

Bogoni, J. A., Graipel, M. E., de Castilho, P. V., Fantacini, F. M., Kuhnen, V. V., Luiz, M. R., . . . Tortato, M. A. (2016). Contributions of the mammal community, habitat structure, and spatial distance to dung beetle community structure. *Biodiversity and conservation*, *25*, 1661-1675.

Hegerl, C., Burgess, N. D., Nielsen, M. R., Martin, E., Ciolli, M., & Rovero, F. (2017). Using camera trap data to assess the impact of bushmeat hunting on forest mammals in Tanzania. *Oryx*, *51*(1), 87-97.

Moran, P. A. (1948). The interpretation of statistical maps. *Journal of the Royal Statistical Society. Series B (Methodological)*, *10*(2), 243-251.

Wilman, H., Belmaker, J., Simpson, J., de la Rosa, C., Rivadeneira, M. M., & Jetz, W. (2014). EltonTraits 1.0: Species-level foraging attributes of the world's birds and mammals. *Ecology*, *95*(7), 2027-2027. doi:https://doi.org/10.1890/13-1917.1
